# Muscle Dystroglycan Organizes the Postsynapse and Regulates Presynaptic Neurotransmitter Release at the *Drosophila* Neuromuscular Junction

**DOI:** 10.1371/journal.pone.0002084

**Published:** 2008-04-30

**Authors:** Laurent Bogdanik, Bérénice Framery, Andreas Frölich, Bénédicte Franco, Dominique Mornet, Joël Bockaert, Stephan J. Sigrist, Yves Grau, Marie-Laure Parmentier

**Affiliations:** 1 CNRS, UMR 5203, Institut de Génomique fonctionnelle, Montpellier, France; 2 INSERM, U661, Montpellier, France; 3 Université Montpellier,1,2, Montpellier, France; 4 European Neuroscience Institute Göttingen, Göttingen, Germany; 5 Institut für Klinische Neurobiologie, Rudolf-Virchow Zentrum, Universität Würzburg, Würzburg, Germany; 6 INSERM, ERI 25, Muscle and Pathologies, Université de Montpellier1, EA 4202, 34295 Montpellier, France; Hospital Vall d'Hebron, Spain

## Abstract

**Background:**

The Dystrophin-glycoprotein complex (DGC) comprises dystrophin, dystroglycan, sarcoglycan, dystrobrevin and syntrophin subunits. In muscle fibers, it is thought to provide an essential mechanical link between the intracellular cytoskeleton and the extracellular matrix and to protect the sarcolemma during muscle contraction. Mutations affecting the DGC cause muscular dystrophies. Most members of the DGC are also concentrated at the neuromuscular junction (NMJ), where their deficiency is often associated with NMJ structural defects. Hence, synaptic dysfunction may also intervene in the pathology of dystrophic muscles. Dystroglycan is a central component of the DGC because it establishes a link between the extracellular matrix and Dystrophin. In this study, we focused on the synaptic role of Dystroglycan (Dg) in *Drosophila*.

**Methodology/Principal Findings:**

We show that Dg was concentrated postsynaptically at the glutamatergic NMJ, where, like in vertebrates, it controls the concentration of synaptic Laminin and Dystrophin homologues. We also found that synaptic Dg controlled the amount of postsynaptic 4.1 protein Coracle and alpha-Spectrin, as well as the relative subunit composition of glutamate receptors. In addition, both Dystrophin and Coracle were required for normal Dg concentration at the synapse. In electrophysiological recordings, loss of postsynaptic Dg did not affect postsynaptic response, but, surprisingly, led to a decrease in glutamate release from the presynaptic site.

**Conclusion/Significance:**

Altogether, our study illustrates a conservation of DGC composition and interactions between *Drosophila* and vertebrates at the synapse, highlights new proteins associated with this complex and suggests an unsuspected trans-synaptic function of Dg.

## Introduction

In muscle fibers, the Dystrophin-glycoprotein complex (DGC) is thought to provide an essential mechanical link between the intracellular cytoskeleton and the extracellular matrix. This complex comprises Dystroglycan subunits (α and β), Dystrophin or Utrophin, sarcoglycans subunits, syntrophin subunits, and dystrobrevin subunits. Dystroglycan (Dg) is a central component of the DGC because it establishes the transmembrane link between Laminins and Dystrophin [1], [2]. Dg is encoded by a single gene (*dag1*) [3], and is expressed as a propeptide that gets cleaved in two fragments: α and β-Dg, which associate non-covalently in mature skeletal muscle. The extracellular Dg α-subunit is heavily glycosylated, and interacts with extracellular Laminin, whereas the transmembrane β-subunit interacts with subsarcolemmal Dystrophin, which itself links the actin network. The maintenance of this structural link provides stability of the sarcolemma, especially upon muscle contraction. In Duchenne muscular dystrophy, mutation and subsequent loss of Dystrophin destabilizes the other DGC members [4], which further leads to mechanical damage of the muscle cell membrane [5], [6]. There are no known dystrophies associated with a mutation in the dystroglycan gene, probably because of the importance of the encoded protein in other cellular types. Indeed, loss of Dg in mice leads to early embryonic death [7]. However, mutations in enzymes responsible for the glycosylation of α-Dg are the cause of congenital myopathies such as the Walker-Warburg syndrome, the muscle-eye-brain disease and Fukuyama-type congenital muscular dystrophy, for review [8], [9].

Most members of the DGC are found to be concentrated at the cholinergic neuromuscular junction. Moreover, mouse studies revealed abnormalities of NMJs lacking DGC components [10]-[15] suggesting a role for a defective neurotransmission in some dystrophies [16], [17]. For example, NMJs without muscle Dg show decreased levels of synaptic Laminin and Utrophin, as well as smaller acetylcholine receptor clusters [17]–[20]. A more general function of the DGC in synaptic transmission is further supported by the localization of DGC components in central brain synapses [21]–[23] and the occurrence of mental retardation in myopathic patients that do not show any major brain structural defect [24]. In this study we focused on the synaptic role of Dg, using the *Drosophila* glutamatergic larval NMJ. This synapse is not only a neuromuscular junction, but also a well-established model to study the development, the structure and the function of a glutamatergic synapse [25]. We showed that Dg is concentrated postsynaptically at this NMJ. We analyzed the status of Dg partners at this synapse and found that 1) like in vertebrates, Dg controls synaptic Laminin and Dystrophin concentration, 2) Dg also controls the amount of postsynaptic 4.1 protein Coracle, the postsynaptic spectrin cytoskeleton, and the relative subunit composition of glutamate receptors, and 3) reciprocally, both Dystrophin and Coracle are required for Dg concentration at the synapse. Finally, electrophysiological analysis shows that loss of muscle Dg leads to a functional defect, i.e. a decrease in presynaptic glutamate release.

## Materials and Methods

### Fly stocks

yw^CS^ flies were used as a control in all experiments with *dg* or *cora* mutants. *dg*-RNAi/+ or UAS-Dg-C/+ flies were used as controls when studying crosses with these transgenes. The 24B-Gal4 line was used for all muscle expression experiments [26]. *dg^323^* and *dg^62^* alleles as well as UAS-Dg-C transgenic flies have been previously described [27]. The genotype of larvae overexpressing Dg-C in our experiments is 24BGal4/UAS-Dg-C. We also used the piggybac insertion *dg^e01554^* [28]. The *dg* directed RNAi construct [27] originally on chromosome 3 was remobilized on chromosome 2 (line n°12), and flies containing the two RNAi transgenes over wild-type chromosomes were used for all immunocytochemistry experiments. We created a UAS-Dg-C-GFP construct using the Gateway system and by inserting the PCR amplified sequence of Dg (LD11619) in ptWG. The Dys-COOH RNAi transgenic flies were described in [29]. The hypomorph *cora^14^* allele was described in [30]. This mutation leads to the replacement of R1607 by a stop codon. The null cora[k08713] allele was analyzed in [31]. The *cora* alleles as well as The P{EPgy2}cora[EY07598] containing stock were obtained from the Bloomington Drosophila Stock Center. Green fluorescent protein or Tubby balancers were used to identify homozygous individuals. All crosses were performed at 25°C except those with RNAi which were set up at 25°C for three days (to allow egg-laying) and transferred at 29°C.

### Generation of polyclonal antibody

The Dystroglycan C-terminal polyclonal antibody (LG5) was raised in New Zealand rabbits by repeated intra dermal injections. Synthetic peptide of the last 7 C-terminal amino acids of Human Dystroglycan (PPPVYPP) was conjugated via a cysteine residue associated to hexanoïc acid to the keyhole limpet hemocyanin and such KLH-linked peptide was used as antigen according to a previously described protocol. The resulting polyclonal antibody was purified and characterized as previously described [32]. Note the Drosophila 7 C-term sequence is PPPVYSP.

### Immunocytochemistry

Larvae were dissected in PBS 1x, EDTA 1mM, and then fixed in fresh 4% paraformaldehyde (Sigma, L'isle d'Abeau, France) for 20 min. for all stainings, apart from GluRIIA and GluRIIC ones. For these stainings, preparations were fixed for 15 min. in Bouin's fixative (Sigma, L'isle d'Abeau, France). Antibody incubations were performed in PBS 1x buffer with 0.3% Triton X100 and 0.2 to 1% BSA. The following antibodies were used: Goat anti-HRP (Sigma, L'isle d'Abeau, France, 1∶1000), mouse monoclonal anti alpha-Spectrin 3A9 (1∶25), mouse monoclonal anti-Dlg 4F3 (1∶100), mouse monoclonal anti-DGluRIIA 8B4D2 (1∶50), mouse monoclonal anti-Fas2 1D4 (1∶20), all four were obtained from the Developmental Studies Hybridoma Bank (University of Iowa, Iowa City, IA); rabbit anti-GluRIIC (gift from A. DiAntonio, 1∶3000) [33], rabbit anti-Dgex8 (gift from M. Schneider, 1∶1000) [29], rabbit anti-Dys (gift from A. Wodarz, 1∶1000) [29], rabbit anti-Laminin (gift from L. Fessler, 1∶1000) [34] and guinea-pig anti-Cora raised against a ∼2KB fragment from the 3′ half of the coding sequence (bp 2193–4225 of cDNA1, see [35]), (gift from R. Fehon, 1∶2500). Alexa 488, Cy3 or Cy5-conjugated anti-rabbit, anti-guinea pig, anti-goat or anti-mouse were obtained from Molecular Probes and Jackson ImmunoResearch, (WestGrove, PA) and used at dilutions ranging from 1∶1000 to 1∶250. Images were acquired with a Biorad 1024 or a Zeiss LSM 510 Meta confocal microscope. Quantification of DGluRIIA and DGluRIIC staining intensities was performed with imageJ. For each channel, a threshold was set and used for all images. The sum of pixel intensities (S) above threshold was calculated for each channel and the ratio of S (DGluRIIA)/S(DGluRIIC) calculated for each image. Student's tests were performed for statistical analysis. 3D views of confocal image stacks were obtained with the Volocity software.

### Co-immunoprecipitation and immunoblot analysis

Drosophila heads from 24B Gal4/+; UAS-Dg-C-GFP/+ flies where homogenized in lysis buffer (50mM Tris pH8, 100mM NaCl, 1% NP40, 1mM EDTA, protease inhibitor cocktail) during 45′ at 4°C. The lysate was centrifuged at 16000 g at 4°C for 20 min. The supernatant was incubated with anti-GFP antibody (A6455, Molecular Probes) O/N at 4°C. Next, we added protein-A conjugated sepharose beads (ProteinA Sepharose CL-4B, GE Healthcare) at 4°C for 3 hours. After incubation, the complex was precipitated at 4000g for 2 min., washed 4 times in lysis buffer, eluted in Laemmli buffer and separated on a 4–12% SDS-Page gel (4–12% GeBagel, Interchim) followed by transfer onto nitrocellulose membrane (Hybond-C, Amersham Biosciences). Membranes were incubated overnight at 4°C with primary antibody diluted in TBS-T (50m M Tris, pH 7.4; 150 mM NaCl and 0.2% Tween 20) containing 5% nonfat dried milk. Dilutions were 1∶10000 for Guinea-pig anti-Cora, 1∶500 for monoclonal anti-alpha Spectrin 3A9 and 1∶1000 for monoclonal anti-Shaggy (4G-1E, Upstate). Membranes were washed three times for 10 min. with TBS-T and incubated with secondary antibodies for one hour at room temperature. Membranes were finally washed three times in TBS-T and signal was detected using SuperSignal West Pico Chemiluminescent Substrate (Pierce).

For analysis of the specificity of the Cora antibody, *Drosophila* larvae from the following genotypes: Canton S, P{EPgy2}cora^EY07598^/+; 24B Gal4/+ and P{lacW}cora^k08713^/cora^14^ were dissected in PBS 1X, EDTA 1mM and then homogenized in lysis buffer (50mM Tris pH8, 100mM NaCl, 1% NP40, 1mM EDTA, protease inhibitor cocktail). Proteins were separated by SDS-PAGE (8%) and transferred electrophoretically onto nitrocellulose membranes (Hybond-C, Amersham Biosciences). Membranes were incubated overnight at 4°C with primary antibodies: anti-Coracle, Guinea-pig, 1∶10000, then anti-Tubulin DM1A, Mouse, 1∶1000, and the procedure continued as described above.

### Quantitative PCR analysis

Dg (CG18250) transcript levels were measured to compare the strength of different *dg* mutant conditions. The following genotypes were tested Canton S, *dg^e01554^* homozygotes, *dg^e01554^*/ *dg^323^* and *dg^e01554^ /dg^62^* transheterozygotes. cDNAs were generated from 1 µg total RNAs treated with DNase I by using random hexamers and Moloney murine leukemia virus reverse transcriptase (LTI). Real-time PCR was done using Applied Biosystems (Courtaboeuf, France) SYBR Green PCR mix according to the manufacturer's instructions. PCR was done as follows: 10 minutes at 95°C followed by 40 cycles: 15 seconds at 95°C, 60 seconds at 60°C. Housekeeping genes used to normalize *dg* expression levels were RPL13, TBP and PGK. Sequences of the primers are: RPL13 5′-AGGAGGCGCAAGAACAAATC and 5′-CTTGCTGCGGTACTCCTTGAG (amplicon 72 nt), TBP 5′-CGTCGCTCCGCCAATTC and 5′-TTCTTCGCCTGCACTTCCA, PGK 5′-TCCTGAAGGTCCTCAACAACATG and 5′-TCCACCAGTTTCTCGACGATCT, Dg couple 1 5′-GAACCGCAGCCGGAAGA and 5′-GGCCTTGCCCGATGTG. And Dg couple 2 5′-GCGACGAAGAGGAGCGCAA and 5′-CCTGAAAGATGACGGGAATACC.

### Electrophysiology

Two-electrode voltage clamp recordings were obtained at 22°C from VLM 6 in segments A2 and A3, of late third instar larvae, as previously described in [36]. All cells selected for analysis had resting potentials between −60 and −70 mV and the input resistance was ≥4 MΩ. Student's tests were performed for statistical analysis.

## Results

### Dystroglycan is a postsynaptic component of the *Drosophila* NMJ

**Figure 1:**
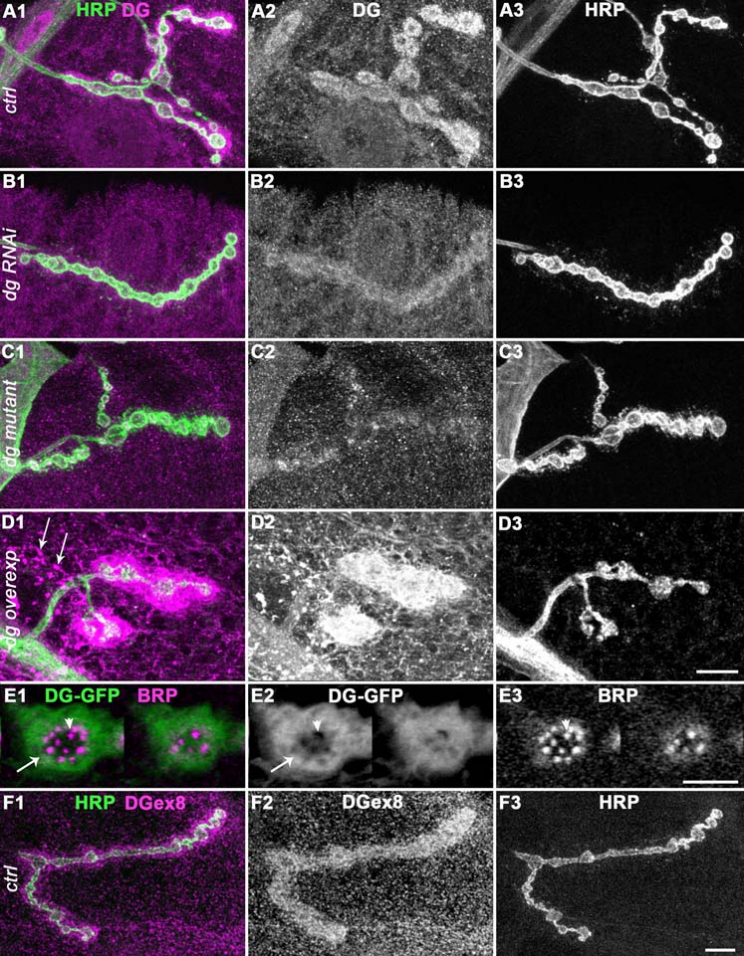
Localization of Dg at the NMJ. A-D) Dg (A2, B2, C2, D2) and HRP (A3, B3, C3, D3) immunoreactivity at the NMJ of muscle 4. Merge of both images with Dg in magenta and HRP in green (A1, B1, C1, D1). The LG5 antibody was used to analyze Dg localization. (A) In wild-type (yw^CS^) flies, Dg concentration is clearly visible at type Ib boutons. Dg immunoreactivity is much larger than motoneuron-specific HRP immunoreactivity, indicating that Dg is present in the muscle cell, i.e. postsynaptically. (B) In larvae expressing *dg*-directed RNAi in the muscle using the 24B Gal4 driver, the NMJ LG5 staining was decreased in intensity. (C) In larvae mutant for *dg* (*dg^e01554^/dg^323^*), the NMJ LG5 staining was more strongly affected. (D) In larvae overexpressing Dg-C isoform in the muscles using the 24B Gal4 driver, a clear increase in LG5 immunoreactivity was observed (laser intensity was decreased to avoid too much signal saturation in this genotype) both at the NMJ, and also in distant patches (arrows). (E) Two serial sections (every 0.7 µm) of a synaptic varicosity of a larva expressing a Dg-C-GFP construct in the muscle cells (with the 24B Gal4 driver). GFP fluorescence (E2) is present in the subsynaptic reticulum (SSR) (arrow) and is partially excluded from the sensus-stricto synapses (arrowhead). Synapses are labelled with the active zone marker Bruchpilot (BRP)(E3). Merge of both images with Dg-GFP in green and BRP in magenta is shown in E1. (F) Dg staining with anti-Dgex8 antibody, specific of the Dg-C isoform (F2) and HRP (F3). The Dgex8 immunoreactivity is similar to the LG5 immunoreactivity (A2). Again, Dgex8 immunoreactivity is much larger than motoneuron-specific HRP immunoreactivity (F1), indicating that Dg-C protein is present at the postsynapse. In all panels, a muscle 4 NMJ is shown. Scale bar is 10 µm in A–D, 5 µm in E and 10 µm in F.

In order to identify proteins of the Dystrophin-glycoprotein complex conserved at the *Drosophila* NMJ, and to study these molecules in a model organism, we undertook a screen of many antibodies directed against mammalian DGC proteins, for a specific staining at the third instar larval NMJ. The LG5 antibody cross-reacted with *Drosophila* Dg (Fig. 1A–D). This antibody was directed against the last 7 C-terminal residues of Human Dystroglycan, which are identical to the *Drosophila* Dg C-terminal residues, apart from the penultimate residue (see Material and Methods). Since the C-terminal residues are common to the three known *Drosophila* Dg isoforms, this antibody certainly recognizes them all. To confirm the data obtained with LG5, we used a second polyclonal antibody, Dgex8, directed against the extracellular mucin-like domain of *Drosophila* Dg [29]. This domain is subjected to significant levels of glycosylation [37], and is encoded by an alternatively spliced exon [27], [29], [38]. It is present only in the Dg-C isoform, and is not required for Dg function in epithelial polarity [29]. Both LG5 and Dgex8 antibodies gave the same result (Fig. 1A and Fig. 1F). They labelled a large area around the HRP-positive varicosities, indicating that some of the staining corresponds to postsynaptically localized Dg. We used transgenic flies that expressed double-stranded RNA directed against *dg* sequence present in all *dg* isoforms mRNAs (dg-RNAi) [27]. Muscle expression of this dg-RNAi construct with the 24B Gal4 driver led to a decrease in the NMJ labelling (Fig. 1B), without significant decrease in the extra-NMJ staining. This indicated that the antibody staining at the NMJ was specific, and that Dg was indeed expressed in the muscles. The presence of Dg at the NMJ was further supported by the analysis of *dg* mutants. Since known null mutants of *dg*, *dg^62^* and *dg^323^* [27] are lethal at the late embryonic, first instar larval stages, we used a hypomorph mutation of *dg*. The Piggybac element insertion PBac{RB}e01554 in *dg* led to a 90% decrease in all *dg* transcripts (Fig. S1). In third instar larvae transheterozygous for *dg^e01554^* and the *dg* null allele *dg^323^*, the Dg synaptic staining was strongly reduced with the LG5 antibody (Fig. 1C) and with the Dgex8 antibody as well (Fig. S1). Finally, when we overexpressed the Dg-C isoform in muscle cells, we observed a large increase in NMJ Dg staining (Fig. 1D), as was already previously reported [39], confirming the synaptic localization of this protein. Overexpressed Dg also aggregated in discrete patches (arrows in Fig. 1D). Altogether, these data show that Dystroglycan, notably the Dg-C isoform containing the mucin-like domain, is endogenously concentrated at the larval NMJ, mainly on the postsynaptic side.

To look in more detail at the fine synaptic localization of Dystroglycan, we expressed a Dg-C-GFP tagged isoform in the muscle. Like the PSD-95 homologue Discs-Large (Dlg) [40], Dg-C-GFP is partly excluded from the postsynaptic densities facing the active zones (Fig. 1E). These latter were labelled with an antibody directed against Bruchpilot (BRP)[36], [41] (Fig. 1). This suggests that Dg-C localization is mainly perisynaptic.

### Dystroglycan controls varicosities Laminin concentration

**Figure 2:**
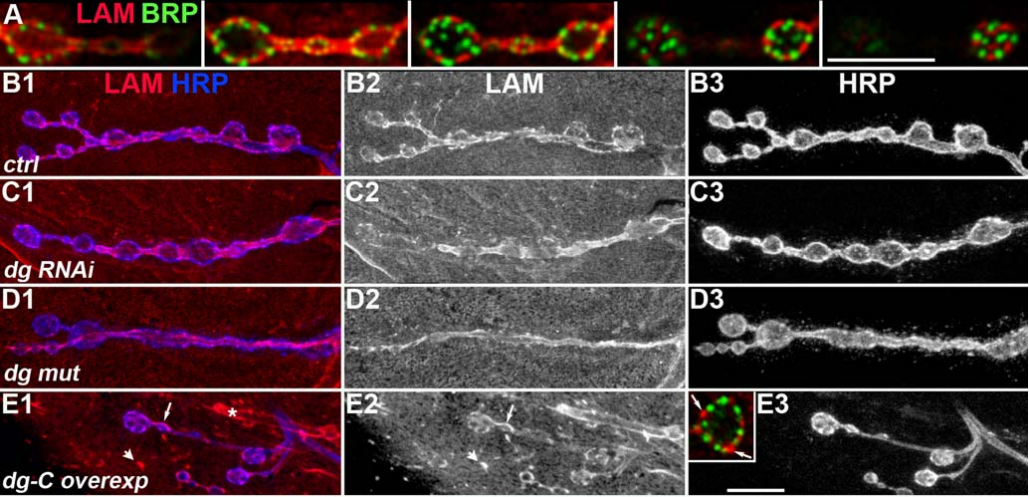
Laminin localization at the *drosophila* NMJ varicosities is influenced by *dg.* A) Serial sections of two adjacent varicosities (every 0.7 µm) labelled for BRP (green) and Lam (red) to analyze Lam localization. There is no colocalization between the active zone marker BRP and the Lam staining, indicating that Lam is mainly perisynatically localized. B–D) Lower magnification of NMJ stained for Lam (B2,C2,D2) and HRP (B3,C3,D3). The merge image is shown in B1,C1,D1 (Lam in red and HRP in blue). (B) In wild-type (yw^CS^) larvae, Lam is present both in varicosities and inter-varicosities connectives. (C) In larvae expressing *dg*-directed RNAi in the muscle using the 24B Gal4 driver (dg-RNAi/+; 24B Gal4/dg-RNAi), the varicosity Lam staining was decreased in intensity, whereas the connective staining remained unchanged. (D) In larvae mutant for *dg* (*dg^e01554^/dg^323^*), the Lam varicosity staining was strongly affected, but not the connective staining. (E) In larvae overexpressing Dg-C isoform with the 24B Gal4 driver, no clear increase in Lam immunoreactivity was observed at the NMJ. However, larger Lam stretches were observed (see arrows in E1, E2 and in the insert showing a double BRP (green), Lam (red) staining of a synaptic bouton in this genotype). In addition Lam patches (arrowhead) appeared in this genotype. N.B. an immunoreactive trachea is visible in E1 and E2 (asterisk). In all panels, a muscle 4 NMJ is shown. Scale bar is 10 µm.

In vertebrates, loss of Dg in muscle cells was reported to suppress Laminin concentration at the cholinergic NMJ [19]. We tested if this was also the case at the *Drosophila* glutamatergic NMJ. First, we investigated whether Laminin was indeed present at the *Drosophila* NMJ. Laminin is a heterotrimer consisting of three chains, A, B1 and B2. We used a polyclonal antibody against Laminin heterotrimer [34]. We could detect some Laminin (Lam) staining at the muscle surface, like in mammalian muscle cells, and could detect Lam immunoreactivity at the NMJ (Fig. 2A). At this level of optical resolution, the NMJ localization of Lam fits with a trans-synaptic localization of this component of the extracellular matrix. Costaining with the active zone marker Bruchpilot revealed that, like Dg, Lam was excluded from synapses (Fig. 2A) and restricted to the perisynaptic zones. Lam was present around varicosities as well as around the inter-varicosities connectives (Fig. 2B). In larvae expressing dg-RNAi in muscles, the Lam content around the varicosities was decreased so that the varicosities were less distinguishable compared to the connectives (Fig. 2C). This situation was similar and even more pronounced in *dg* mutants (Fig. 2D). We also analyzed the consequences of Dg overexpression in the muscle cell on the amount and localization of Lam. We used the Dg-C isoform, containing the mucin domain, which is supposed to play a critical role in Lam binding of Dg [29], [42]. Lam patches, never observed in control conditions, appeared extrasynaptically and colocalized with Dg-C-GFP (Fig. S2). This indicated that the Dg-C isoform could indeed recruit Lam in the muscle cell. At the NMJ, the pattern of Lam localization was more irregular, with some perisynaptic areas larger, and more intense in Lam immunoreactivity, compared to a more homogenous situation in controls (Fig. 2E). Altogether, these data show that Laminin localization around varicosities is partly dependent on Dg. We then tested the interaction between Dg and its second well-known partner, Dystrophin, at the synapse.

### Dystroglycan controls postsynaptic Dystrophin concentration

**Figure 3:**
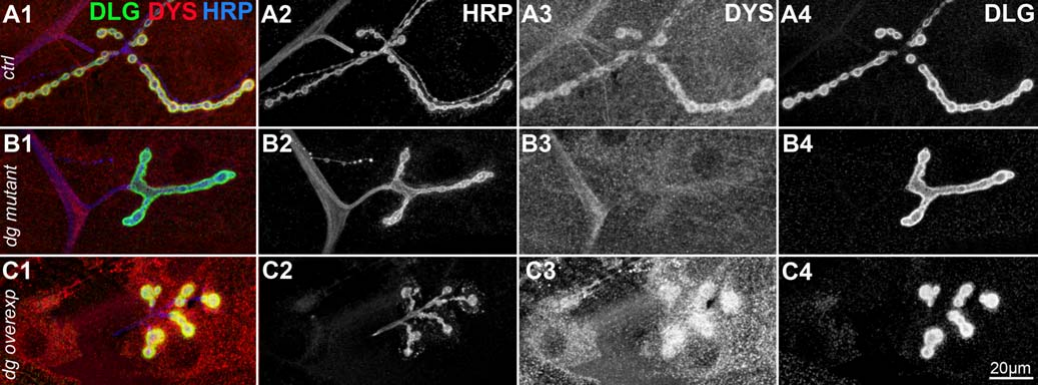
Dg controls synaptic Dys localization. Triple staining for HRP (blue), Dys (red) and Dlg (green) in control (yw^CS^) (A), *dg* mutant (B) and Dg-C overexpressing (C) larvae. Merge images are shown in A1, B1, C1. Single channel stainings are shown in A2, B2, C2 for HRP, A3, B3, C3 for Dys and A4, B4, C4 for Dlg. In *dg^e01554^/dg^323^* mutant larvae, the Dys staining (B3) is almost absent compared to wild-type larvae (A3), although the Dlg staining is still present (compare A4 and B4). When Dg-C is overexpressed with the 24B Gal4 driver, Dys staining is strongly enhanced around HRP positive boutons, and also in the entire muscle cell (compare C3 and A3). In all panels, a muscle 4 NMJ is shown. Scale bar is 10 µm.

Dg was shown to be partly responsible for the synaptic concentration of Utrophin at the vertebrate cholinergic NMJ [18]. In *Drosophila*, there is only one gene homologous to utrophin and dystrophin genes, called *dystrophin* [43]. This gene encodes for different protein isoforms. Large isoforms are concentrated postsynaptically at the NMJ [44]. To test whether synaptic Dystrophin (Dys) localization was dependent on Dg, we used an antibody directed against a C-terminal sequence of Dys, which detects all known Dys isoforms [29]. As previously described with an antibody directed against large isoforms of Dys, we observed a postsynaptic localization of Dys at the NMJ (Fig. 3A). *Dg* mutants displayed a strongly reduced Dys staining (Fig. 3B1-3). This reduction was not due to a general absence of the postsynaptic apparatus since postsynaptic marker Discs-Large (Dlg) staining was present in these mutants (Fig. 3B4). This indicates that Dg is required for the normal postsynaptic Dys localization. To test whether Dg was also sufficient for this Dys localization, we overexpressed postsynaptically Dg-C. In these NMJs, Dystrophin immunoreactivity was strongly enhanced around synaptic boutons (Fig. 3C), indicating that postsynaptic Dg can indeed recruit or stabilize Dys. Altogether, these data show that Dg controls Dys concentration at the glutamatergic NMJ.

**Figure 4:**
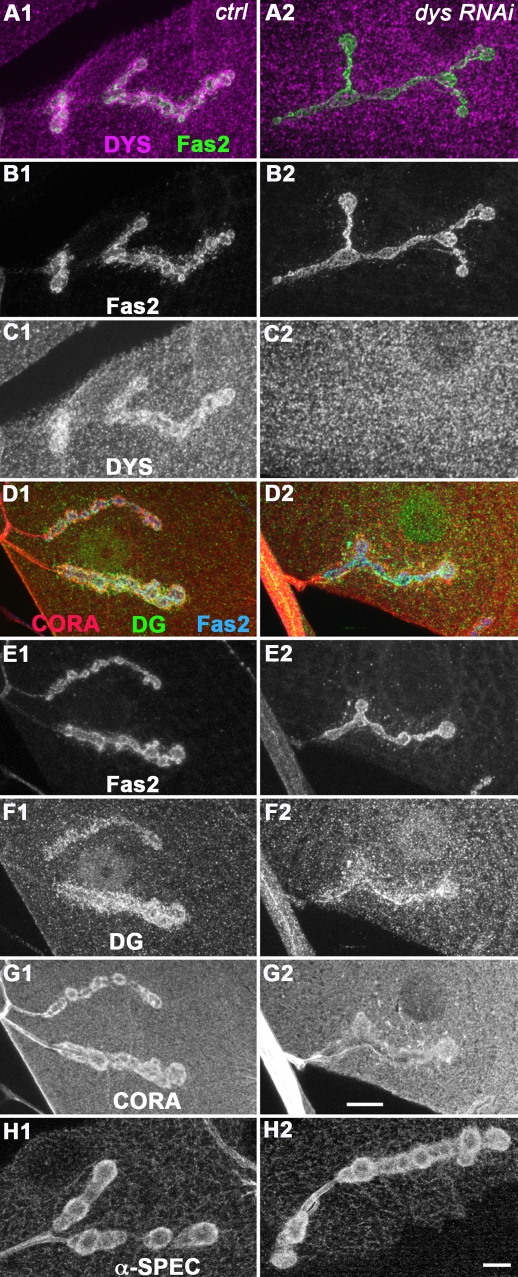
Dys controls Dystroglycan postsynaptic concentration. (A–C) Double staining for Fas2 (A green, B), Dys (A magenta, C) on control (1) and UAS-dys-RNAi flies crossed with 24B Gal4 (2). In larvae expressing the dys-RNAi (dys-RNAi/+; 24B Gal4/+), there is almost no Dys immunoreactivity detectable at the NMJ. (D–G) Triple staining for Fas2 (D blue, E), Dystroglycan (D green, F) and Coracle (D red, G) in the same genotypes. Dg postsynaptic labelling is reduced in absence of postsynaptic Dystrophin. Coracle immunoreactivity is also reduced, but to a much lower extent compared to the loss of postsynaptic Dg. (H) alpha-Spectrin immunostaining on the same genotypes. Scale bar is 10 µm. In all panels, a muscle 4 NMJ is shown.

Dystrophin is known to be required for Dg sarcolemmal localization in vertebrate muscles [4]. We wondered whether Dys was also required for normal Dg localization at the *Drosophila* NMJ. To test this hypothesis, we used RNAi interference to target all Dystrophin isoforms in the muscle [29]. Expression of the RNAi transgene led to a very strong decrease in postsynaptic Dys immunoreactivity (Fig. 4A–C). This lack of postsynaptic Dystrophin was accompanied by a decrease in postsynaptic Dg (Fig. 4D, F), although not a complete loss of Dg. Hence, at the *Drosophila* NMJ, Dys partially controls Dg synaptic concentration. Altogether, our data illustrate the presence of a synaptic Lam/Dg/Dys complex, like in vertebrate neuromuscular junctions. What can be the functional consequences of the loss of this complex in absence of Dg? At the cholinergic NMJ in mammals, Dg is involved in synapse function through rapsyn-mediated anchoring of the nicotinic acetylcholine receptors [19], [45], [46]. We wondered whether Dg is also required for the clustering of glutamate receptors and if so, through which cytoplasmic scaffold. We first looked at the synaptic protein Coracle, reported to control some glutamate receptor subunits localization at the *Drosophila* NMJ [31].

### Dystroglycan also controls intracellular Coracle NMJ concentration

**Figure 5:**
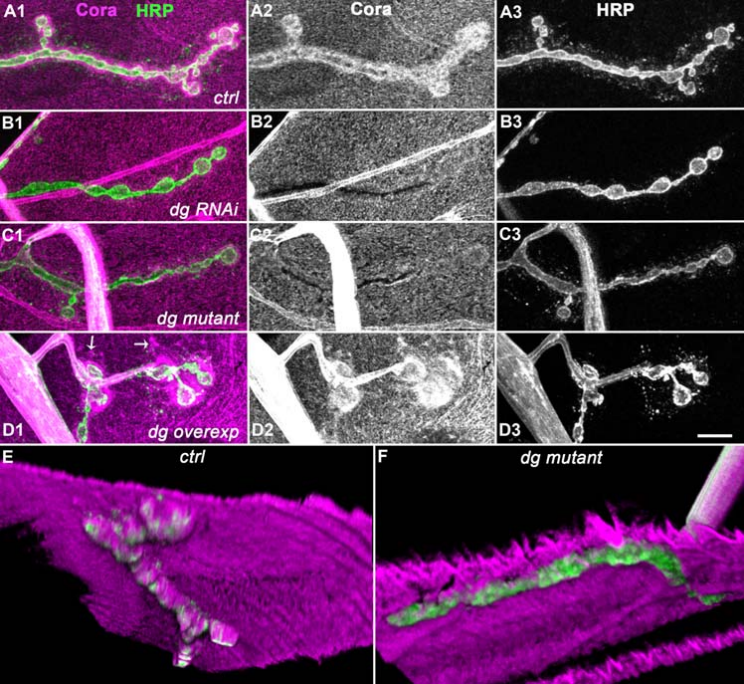
Dg controls postsynaptic Cora concentration. Double staining for Cora (Magenta) and HRP (green)(1) in (A) control (yw^CS^) larvae, (B) larvae expressing muscle *dg*-RNAi (dg-RNAi/+; 24B Gal4/dg-RNAi), (C) *dg^e01554^/dg^323^* mutant larvae and (D) larvae overexpressing Dg-C in muscles (24B-Gal4/UAS-DgC). Arrows indicate patches of Cora protein. Single stainings for Cora and HRP are shown respectively in (2) and (3). In all panels, a muscle 4 NMJ is shown. Scale bar is 10 µm. 3D views of the preparations shown in A1 and C1 are shown in E and F: we look at the NMJ from the inside of the muscle cell. Cora is in magenta and HRP in green.

Coracle (Cora) is the *Drosophila* 4.1 protein homologue. It has been shown to be concentrated at the NMJ and to control the amount of GluRIIA subunit in postsynaptic glutamate receptors [31] in late embryos and first instar larvae. The *cora* gene encodes four protein isoforms (ranging from 699 to 1698 amino acids) [35]. In Chen et al. [31], the 9C monoclonal antibody directed against the FERM domain of all Cora isoforms [35] was used, and no clear Cora immunoreactivity could be detected at the NMJ of late larval developmental stages. Here, we demonstrated that in third instar larvae, Cora was concentrated around the NMJ (Fig. 5A), using a polyclonal guinea-pig antibody directed against a sequence present only in large Cora isoforms [35]. We tested the specificity of the stainings we obtained in flies with genetically altered levels of Cora protein, where the intensity of the staining should parallel the amount of protein. We used the *cora^14^* hypomorph mutant [30] and the null mutation cora*^k08713^* [31] as well as flies overexpressing *cora*. In cora*^14^*/cora*^k08713^* third instar larvae, the NMJ Cora staining was strongly reduced, indicating that this staining was specific and that Cora was actually present postsynaptically in the domain rich in subsynaptic reticulum (SSR) (Fig. S3). We also overexpressed Cora in muscles, using the 24B Gal4 driver and the P element insertion P{EPgy2}cora[EY07598] upstream of the translation start of all Cora isoforms. This produced an increased staining in the SSR as well as in the whole sarcolemma (Fig. S3). These data further confirmed the ability of this antibody to recognize Cora. We observed that in larvae expressing a *dg* directed RNAi and in *dg* mutants, the synaptic concentration of Cora was markedly affected (Fig. 5 B,C,E,F). No clear change in intensity was observed for the sarcolemmal immunoreactivity. These data showed that Dg was required for the NMJ localization of Cora. We further tested whether Dg was also sufficient to concentrate Cora at the synapse, by overexpressing Dg-C in the muscle. This led to a very large increase in the amount of postsynaptic Cora (Fig. 5D), but not to an overall homogenous increase of Cora immunoreactivity at the muscle surface. However, many cytoplasmic Cora clusters could be found, in a way similar to Laminin clusters when Dg-C was overexpressed (arrows in Fig. 5D). In Dg-C-GFP overexpressing larvae, the Cora patches were localized at positions where Dg-C-GFP could be found, exactly like the Lam patches (Fig. S2). This further confirms the ability of Dg-C to recruit the Cora protein. Altogether, these data show that Dg controls synaptic Cora localization.

### Dystroglycan controls the abundance of Spectrin cytoskeleton at the NMJ

**Figure 6:**
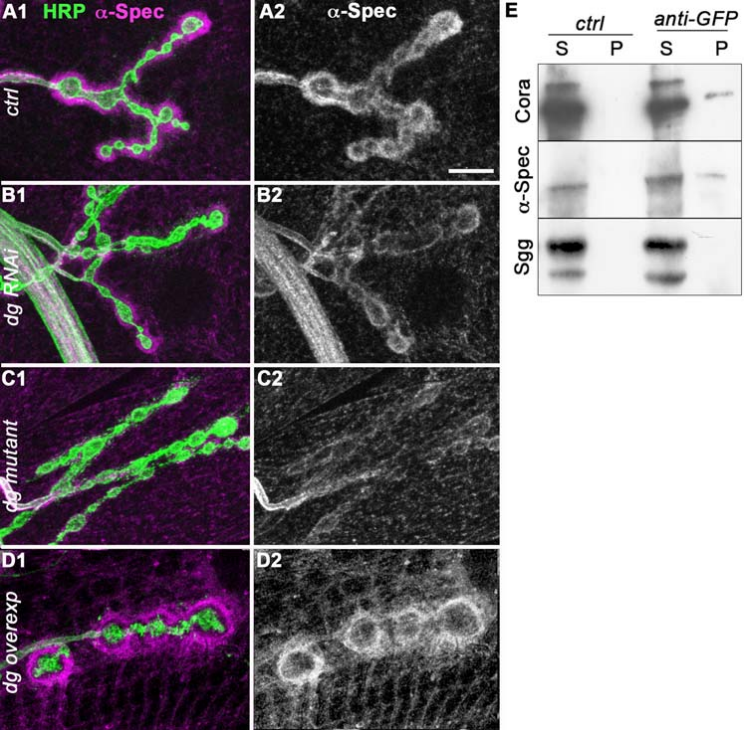
Dg controls postsynaptic Spectrin concentration and both Cora and Spectrin co-immunoprecipitate with Dg. Double staining for alpha-Spectrin (Magenta) and HRP (green)(1) in (A) control (yw^CS^) larvae, (B) larvae expressing muscle *dg*-RNAi (dg-RNAi/+; 24B Gal4/dg-RNAi), (C) *dg^e01554^/dg^323^* mutant larvae and (D) larvae overexpressing Dg-C isoform in the muscles (24B-Gal4/UAS-DgC). Single stainings for alpha-Spectrin are shown in (2). Scale bar is 10 µm. (E) Co-immunoprecipitation was performed with a polyclonal anti-GFP antibody on protein extracts from flies expressing Dg-C-GFP. S corresponds to the supernatant and P to the pellet. Cora and alpha-Spectrin co-immunoprecipitate with Dg-C-GFP, but not Shaggy (Sgg), a cytoplasmic protein kinase.

The 4.1 proteins are described as spectrin-actin binding proteins [47]. The prototypical 4.1 protein is the erythrocytal 4.1 protein (4.1R), which is essential for the submembranous actin-spectrin cytoskeleton. The protein domain of 4.1R protein responsible for this interaction has been delineated and is called the SAB domain. This domain is not conserved in the *Drosophila* homologue Cora [35], which is probably not able to bind actin and spectrin. The spectrin cytoskeleton is composed of heteromers of alpha and beta spectrin subunits. Previous studies have shown that this cytoskeleton is also present at the NMJ [48]. Since we observed a clear colocalization of Cora and Spectrin at the NMJ postsynaptic side (Fig. S3), we looked whether loss of Cora in the *dg* mutant conditions influenced the synaptic spectrin cytoskeleton. In *dg* loss of function conditions, there was a decrease in the postsynaptic staining intensity of the spectrin cytoskeleton, as assessed with an anti-alpha Spectrin antibody (Fig. 6B, C) compared to the wild-type situation (Fig. 6A). However, this cytoskeleton was still clearly visible. In addition, Dg postsynaptic overexpression led to an increase in postsynaptic alpha-Spectrin immunoreactivity, which was not completely penetrant (Fig. 6D). Hence, our data show that Dg controls postsynaptic Cora concentration, and, to a lesser extent, the postsynaptic spectrin cytoskeleton.

To address whether Cora and Spectrin proteins belonged to the protein complexes interacting with Dg, we performed immunoprecipitation of a Dg-GFP protein expressed in muscles. Both Cora and alpha-Spectrin proteins co-immunoprecipitated with Dg-GFP, whereas a control protein like the GSK-3 homologue Shaggy (Sgg) did not co-immunoprecipitate (Fig. 6E). In conclusion, Cora and alpha-Spectrin are part of a protein complex including Dg.

### Dg effect on Cora and alpha-Spectrin is not merely a consequence of the loss of Dystrophin

The most parsimonious way to explain how Dg can affect Cora and alpha-Spectrin NMJ concentration is: Dg controls the F-actin cytoskeleton via Dystrophin; Coracle as well as alpha-Spectrin, known to be actin binding protein, are delocalized when the postsynaptic F-actin cytoskeleton is disrupted in absence of Dystrophin in the *dg* loss of function condition. To test this hypothesis, we tried to look at the postsynaptic F-actin cytoskeleton with rhodamine-phalloidin staining, but could not reliably see its size due to the underlying actin-myosin contractile apparatus. Still, some postsynaptic F-actin cytoskeleton could be observed in *dg* mutants (data not shown). We thus tested whether the loss of Dystrophin also led to a decrease in Cora and alpha-Spectrin postsynaptic immunoreactivity. We performed this experiment, keeping in mind that the loss of Dystrophin also affected Dg immunoreactivity (Fig. 4). We could see that an almost complete loss of NMJ Dystrophin obtained when expressing a Dys RNAi construct always had smaller effects on Cora and α-Spectrin immunoreactivity compared to a loss of Dg function, which affected only partially Dys staining (compare Fig. 4 and Fig. 5). These results suggest that Dg controls postsynaptic Cora and alpha-Spectrin concentration independently of Dystrophin (Fig. S4).

### Coracle reciprocally controls Dg localization

**Figure 7:**
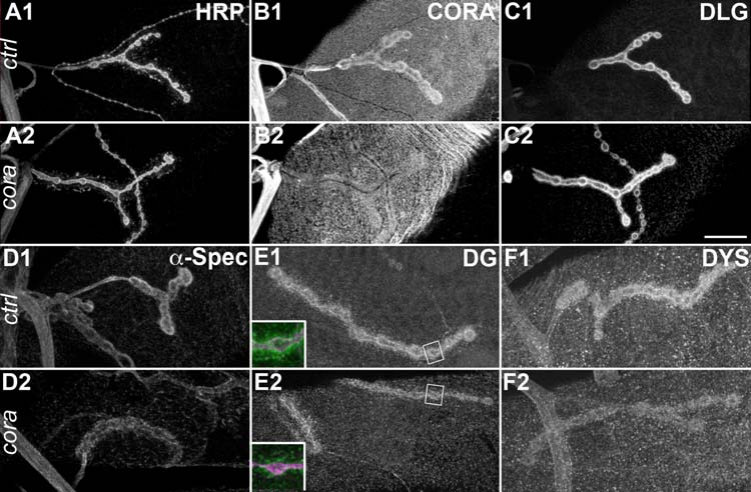
Cora controls Spectrin, Dystroglycan, Dystrophin but not Dlg postsynaptic concentration. (A–C) Triple staining for HRP (A), Cora (B) and Dlg (C) in control (yw^CS^) (1) and *cora^14^*/*cora^k08713^* larvae (2). Cora concentration at the NMJ is largely decreased in the cora mutant, whereas synaptic HRP and Dlg stainings show the same signal intensity. This indicates that Cora synaptic loss in the *cora* mutant is not the direct consequence of a total disruption of NMJ structure. (D–F) Stainings for alpha-Spectrin (D), Dgex8 (E) and Dystrophin (F) in control (1) and *cora^14^*/*cora^k08713^* larvae (2). (D) In *cora* mutants, alpha-Spectrin postsynaptic concentration decreases, but does not completely disappear, like in *dg* mutants. (E) Dystroglycan staining appears thinner in *cora* mutants. Inserts show higher magnification of a synaptic bouton with presynaptic HRP in magenta and Dg staining in green. The thinner appearance of the NMJ in *cora* mutants is due to a decrease in postsynaptic Dg staining compared to WT larvae. (F) In *cora* mutants, Dys labelling is reduced, but is still visible, contrarily to *dg* mutants, in which it disappears more strongly. Scale bar is 20 µm.

If Dg controls in parallel Dys and Cora synaptic concentration, and Dys controls reciprocally Dg, we wondered whether Cora also played such a reciprocal function on Dg. We looked at the Dg NMJ staining in the cora*^14^*/cora*^k08713^* mutant. This mutant reproduced the *dg* phenotype at the NMJ, in that postsynaptic Cora concentration was markedly affected (Fig. 7B), although presynaptic HRP staining (Fig. 7A) and postsynaptic Dlg staining (Fig. 7C) did not change intensity. In this *cora* hypomorph mutant, postsynaptic Dg staining was reduced (Fig. 7E). This indicated that Cora controlled Dg postsynaptic localization and that the functional interactions between Dg and Cora were reciprocal. Associated with the postsynaptic Dg loss, we could observe, as expected, a decrease in alpha-Spectrin immunoreactivity (Fig. 7D) and in Dystrophin staining (Fig. 7E).

### Dg influences the amount of GluRIIA subunits in postsynaptic glutamate receptor clusters

**Figure 8:**
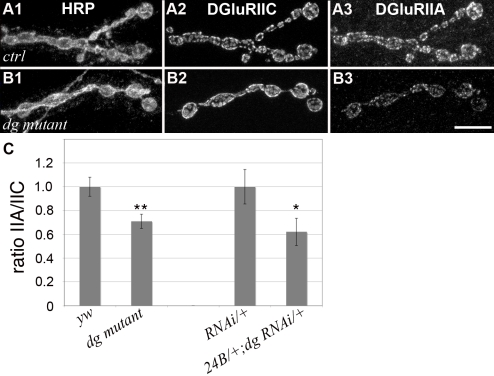
Dg influences glutamate receptor subunit composition. Triple staining for HRP (1), DGluRIIC (2) and DGluRIIA (3) in control (yw^CS^) (A) and *dg^e01554^/dg^323^* mutant larvae (B). DGluRIIA immunoreactivity is reduced in the *dg* mutant whereas DGluRIIC and HRP immunoreactivities are unchanged. (C) Quantification of the ratio of DGluRIIA versus DGluRIIC staining intensities (n = 6 for yw^CS^ and *dg* mutant, and n = 8 for dg-RNAi/+ and dg-RNAi/+; 24B Gal4/+). (* p<0.5; ** p<0.01). Error bars represent SEM.

The Drosophila NMJ is a glutamatergic synapse. The postsynaptic glutamate channel receptors are thought to be composed of four subunits: GluRIIC, IID, IIE and either the GluRIIA or GluRIIB subunit [33], [49]–[52]. Since Cora was shown to control the amount of GluRIIA, and since the amount of postsynaptic Cora was largely reduced in *dg* mutants, we expected to see a decrease in the amount of GluRIIA subunit present in the postsynaptic glutamate receptors. We quantified the immunoreactivity for GluRIIA compared to the immunoreactivity for GluRIIC and could indeed see a significant decrease in the relative amount of GluRIIA subunit (Fig. 8), although we could never see a complete loss of this subunit. These data show that Dg influences the relative subunit composition of glutamate receptors at the NMJ. We then analyzed electrophysiologically the consequences of the loss of postsynaptic Dg.

### Loss of postsynaptic Dystroglycan leads to a decrease in synaptic quantal content

**Figure 9:**
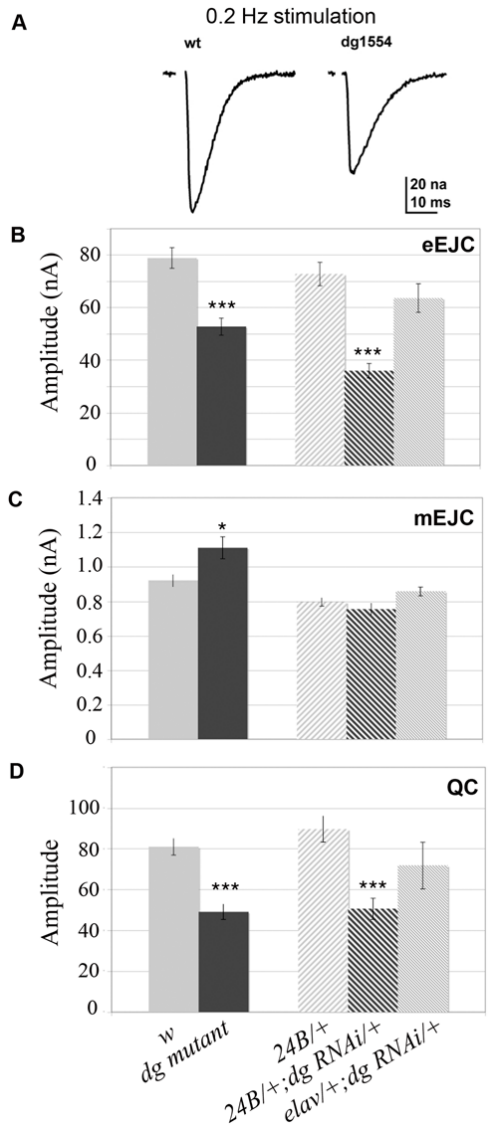
NMJ electrophysiological defects due to the loss of *dg* function. A) Example traces of evoked postsynaptic currents (EJCs) from NMJs of wild type (w^1^) and *dg* mutant *dg^e01554^* larvae. B) Mean EJC amplitude for wild type (w^1^ larvae), *dg* mutant *dg^e01554^*, Canton S crossed with 24B Gal4 (24B Gal4/+), dg-RNAi crossed with 24B Gal4 (dg-RNAi/+;24B Gal4/+) and dg-RNAi crossed with elavC155 Gal4 (elav Gal4/+; dg-RNAi/+). B) mini EJC amplitudes and C) quantal content for the same genotypes. The number of measured larvae is indicated within each histogram bar. (* p<0.5; *** p<0.001). Error bars represent SEM.

We performed intracellular, two-electrode voltage clamp recordings from muscle 6 of segments A3 at the larval NMJs of WT and *dg^e01554^* hypomorphic mutants, or of 24B/+ and dg-RNAi/+; 24B/+ larvae (Fig. 9). In *dg* mutants, or in flies expressing dg-RNAi in muscles, the evoked junctional currents (EJCs) were decreased by approximately 40% (*w* control: 73.95±3.47 nA, n = 11; *dg* mutant: 53.86±3.87 nA, n = 10; 24B/+ control: 72.80±4.45 nA, n = 10; dg-RNAi/+; 24B/+: 36.02±2.75 nA, n = 11) (Fig. 9A and 9B). This effect could be due either to a decrease in the sensitivity of the postsynaptic glutamate receptor field, which should be reflected in a decrease of miniature junctional currents amplitudes (mEJCs). On the other hand, the number of vesicles released per action potential (“quantal content”) could be reduced. mEJCs amplitude was not diminished when Dg was decreased (*w* control: 0.92±0.034 nA, n = 11; *dg* mutant: 1.11±0.064 nA, n = 10; 24B/+ control: 0.80±0.024 nA, n = 9; dg-RNAi/+; 24B/+: 0.76±0.036 nA, n = 10) (Fig. 9C). On the contrary, there was a slight enhancement in the *dg* mutant, but not reproduced in dg-RNAi larvae. However, estimation of quantal content (EJC/mEJC) revealed a reduction of about 40% in *dg* mutant and dg-RNAi larvae (*w* control: 81.1±4.1, n = 11; *dg* mutant: 49.2±3.8, n = 10; 24B/+ control: 89.8±6.4, n = 9; dg-RNAi/+; 24B/+: 50.6±5.2, n = 10) (Fig. 9D). Hence, postsynaptic Dg seems to be able to positively control the amount of presynaptic vesicles released upon stimulation. We further verified that the effect observed in dg-RNAi/+; 24B/+ flies was not due to a non specific presynaptic expression of the RNAi, by comparing this result with flies expressing the RNAi in neurones with the elav-Gal4 driver. In elav/+; dg-RNAi/+ larvae, the evoked junctional currents (EJCs) were only slightly decreased compared to control genotypes (elav/+; dg-RNAi /+: 63.65±5.41 nA, n = 9) (Fig. 9B). In these larvae, the mEJCs amplitude was not affected (0.86±0.025 nA, n = 5) (Fig. 9C) and the quantal content was not significantly diminished compared to both the w and the 24B/+ control genotypes (71.86±11.40 nA, n = 5) (Fig. 9D). Since larvae expressing dg-RNAi in motoneurones do not show as strong a phenotype as larvae expressing dg-RNAi in muscles, this indicates that postsynaptic Dystroglycan controls presynaptic vesicle release.

## Discussion

### Synaptic localization of Dg

The widely accepted hypothesis about the function of the DGC complex is its protective role in the sarcolemma against muscle contraction induced size changes. Here we analyzed the synaptic function of a core member of the DGC, Dystroglycan. We showed that *Drosophila* Dg was concentrated at the NMJ, and that most Dg immunoreactivity at the NMJ was postsynaptic. We also showed that a proportion of synaptic Dg contained the mucin-like domain (MLD), which is the most heavily glycosylated domain in vertebrate Dg. Haines et al reported recently that the MLD containing *Drosophila* Dg isoform was indeed glycosylated [39]. Thus, like the vertebrate cholinergic NMJ, the *Drosophila* NMJ is enriched in Dg, and notably in glycosylated forms of this protein. These data are in accordance with the previously described concentration of Dystrophin at the *Drosophila* NMJ [44], suggesting the presence of all DGC members at the postsynapse.

It is possible that the NMJ defects observed in the *dg* mutants used in this study are a consequence of a general muscle dysfunction, due to the loss of Dg at extrasynaptic sites. Indeed, muscle dysfunction has been observed in *dg* null mutants that are lethal at the embryonic and first instar larval stage [39]. However, the mutants analyzed here are hypomorphs and the allelic combination used, *dg^e01554^/dg^323^*, is viable. The larvae crawl, pupate and give rise to fertile adults, which do not show any wing position phenotype corresponding to flight muscle degeneration. Although we cannot rule out that there are some subtle muscle defects at extrasynaptic sites, our data illustrate that synaptic electrophysiological and morphological defects are already present in these mild loss of function conditions.

### Laminin-Dg-Dys complex is conserved at the *Drosophila* glutamatergic NMJ

A function of the *lanA* gene, encoding a Laminin A subunit, in stabilizing the initial motoneuron/muscle contact during synaptogenesis was published earlier [53]. Here, we show that Laminin is still present during late larval stages, and that it is concentrated around synapses in varicosities. Our data indicate that, like in mice where Dg is required for synaptic Utrophin, Laminin α5 and Laminin γ1 concentration [13], *Drosophila* Dg controls synaptic Laminin and Dystrophin concentration. In addition, Dystrophin was known to be required for Dg sarcolemmal localization in vertebrate muscles [4], and both Dystrophin and Utrophin account for part of the clustering of Dg at the NMJ [54], [55]. Here, we show that Dystrophin also controls synaptic Dg concentration. Thus the interdependence between Laminin, Dg and Dystrophin at the NMJ seems to be conserved phylogenetically. Importantly, in dystrophin/utrophin double mutants, a significant amount of Dg remains at the synapse, indicating that other proteins control, in parallel, its synaptic localization [54], [55]. Our observations indicate that, similarly, the Utrophin-Dystrophin homologue in flies does not account for the whole synaptic localization of DG, and we identify Coracle as a new, additional synaptic anchor for Dg.

### The 4.1 protein Cora at the NMJ

Looking for any new potential partners of Dg, we studied Cora localization in late larval stages at the NMJ. A previous report described a function for Cora in early larval stages [31], but did not show any clear synaptic localization of Cora in late larval stages, using a monoclonal antibody [35] recognizing all Cora isoforms. Instead, a strong immunoreactivity in NMJ associated glial cells was reported. We used a polyclonal antibody recognizing only the large Cora isoform [35]. With this antibody, we had no immunoreactivity in any NMJ associated glial cell, but we could easily detect a postsynaptic concentration of Cora, which partially disappeared in a *cora* hypomorph mutant, and was increased when Cora was overexpressed in the muscle. These data indicated that the observed staining was indeed Cora. The Localization of protein 4.1 members in vertebrate muscle fibers is not well documented. It has been shown that protein 4.1R isoforms were indeed present in the muscle cells, notably at the cell periphery (probably the sarcolemma) [56]. Interestingly, in DMD patients, the peripheral localization of protein 4.1R isoforms was lost, although the sub-sarcolemmal spectrin cytoskeleton was still present [56]. This set of data already indicated that protein 4.1 sarcolemmal localization was dependent on the DGC complex. Here, we show that this is the case at the NMJ, and that Dg is the principal component involved in Cora localization, since loss of postsynaptic Dys gave much weaker phenotypes compared to loss of postsynaptic Dg. In addition, we show that Cora co-immunoprecipitates with Dg, indicating the presence of the two proteins in the same complex, although further biochemical analysis will be required to assess whether they interact directly or indirectly.

Unexpectedly, we observed that Cora was required for the normal postsynaptic localization of Dg and, to a lesser extent, of Dys. This result was observed using a hypomorph *cora* mutant in which the C-terminal domain is partially deleted. In this mutant, synaptic amount of Cora was strongly reduced (Fig. 7 and Fig. S3). Further structure-function studies will be required to understand 1) which domain of Cora is required for its synaptic localization and for its interaction with Dg, 2) which part of Dg C-terminal tail is involved in Cora interaction. Previous studies have shown that the juxtamembrane region of the C-terminal Dg tail was interacting with Ezrin, a protein containing a FERM domain, like Cora [57], [58]. It is possible that the same Dg domain interacts with Cora.

Since Cora was known to control synaptic GluRIIA abundance [31], an expected consequence of the loss of synaptic Cora in *dg* mutant NMJ was a reduction in the amount of GluRIIA subunit at the NMJ. We found such a reduction, but to a mild degree (relative quantification to the amount of GluRIIC subunit had to be done to decrease the effect of preparation variance). This small effect may be due to the fact that *dg*-induced reduction of synaptic Cora is not as strong as a complete *cora* loss of function, which was the situation analyzed originally. The small effect observed on DGluRIIA probably explains why there was no change in the amplitude of mEJCs in *dg* loss of functions. Indeed, DGluRIIA is the dominant subunit compared to DGluRIIB and a significant loss of DGluRIIA should lead to a decrease in mini amplitude [51].

### Dystroglycan and the spectrin cytoskeleton

Loss of synaptic Dystroglycan resulted in a clear decrease in postsynaptic spectrin cytoskeleton, as assessed with alpha-Spectrin immunoreactivity. Although the spectrin defect may be a consequence of the loss of synaptic Cora, a more direct interaction between Dg and the spectrin cytoskeleton remains a possibility. Hence, the link between Dg, Cora and spectrin cytoskeleton remains to be further defined. The postsynaptic spectrin cytoskeleton was shown to play a role in the repartition of postsynaptic receptor fields [48]. Indeed, loss of postsynaptic immunoreactivity for both alpha and beta-Spectrin led to a disorganization of postsynaptic receptor fields. We looked for such a defect in the dystroglycan loss of function conditions, but could not find any. This is probably due to the fact that the loss of spectrin immunoreactivity in these mutants was not complete.

### Presynaptic glutamate release defect in postsynaptic *dg* deficient larvae

We demonstrated that Dg played a functional role in neuromuscular synaptic transmission. Indeed, the glutamate release was decreased by approximately 40% in absence of muscle Dg. The main specificity of the insect NMJ, compared to the vertebrate NMJ is the presence of glutamate as a neurotransmitter instead of acetylcholine. Hence, these synapses are not only NMJ models, but also models of glutamatergic synapses, which are by far the most frequent synapses found in the vertebrate brain. A previous study of Dg function in brain synapses illustrated an alteration of LTP in DG^−CNS^ mice, but no modification of the amplitude of synaptic responses evoked by low frequency stimulation of Schaeffer collaterals, and no changes in paired-pulse facilitation [59]. Here, we could detect a reduced synaptic response at low frequency, indicating a function of Dg in basal glutamatergic synaptic transmission.

One surprising result in our electrophysiology experiments was the fact that defects in quantal content of the *dg* mutant were also present, with the same intensity in flies expressing a 24B Gal4 driven dg-RNAi. This indicated that loss of postsynaptic Dg led to a functional change in the other synaptic compartment, the presynapse. Such a presynaptic effect associated with postsynaptic modifications is not new, since the NMJ function displays homeostasis, and that decrease in postsynaptic responsiveness is often associated to increase in neurotransmitter release and vice-versa in order to maintain constant EJCs [60]. The molecular mechanisms involved in this homeostatic control are largely unknown. Here, in *dg* mutants, homeostatic control is likely absent since mini amplitude (receptor field) is not altered in absence of postsynaptic Dg, but glutamate release is modified. This suggests that Dg-deficient muscles inappropriately signals to the presynaptic release machinery. A similar trans-synaptic effect of loss of muscle Dystrophin onto presynaptic quantal content was observed [44]. What can be the mechanisms involved? One possibility is that postsynaptic Dg directly controls the levels of synaptic ECM molecules such as Laminin. These proteins, by interacting with presynaptic receptors, would affect the structure of the presynapse, e.g. the amount, size or molecular composition of active or periactives zones. This hypothesis is strongly supported by the finding in mouse, that a synaptic Laminin-calcium channel interaction organizes active zones in motor nerve terminals [61]. Another presynaptic Laminin receptor could be the synaptic vesicle protein SV2 [62]. Looking for modifications at the presynapse, we did not detect any obvious change in the number and size of active zones, using Bruchpilot immunoreactivity as a marker (unpublished observations) and did not detect any modification in the immunoreactivity of the periactive zone marker Fas2 (unpublished observations). Still, the regulation of synaptic Laminin by Dg that we demonstrate here, together with the presynaptic electrophysiological phenotype we observed, make the hypothesis of Laminin bridging postsynaptic Dg and the presynapse, at least in periactive zones, very likely.

These findings, i.e. the new components of a Dystroglycan complex, as well as the unexpected trans-synaptic role of Dg pave the way for understanding the role of the DGC in the formation, maintenance and plasticity of glutamatergic synapses.

## Supporting Information

Figure S1 dg gene structure, dg mutants and qRT-PCR analysis A) Gene structure of dg gene compiled from flybase data and [27]. The two null alleles dg323 and dg62 are deletions comprising the first exon. The Piggybac element PBac{RB}e01554 is localized within the ninth exon, subjected to alternative splicing. B) To analyze how the Piggybac element altered dg transcription, we perfomed qRT-PCR experiments with couples of oligonucleotides designed against sequences common to all transcripts, either in the extracellular domain (couple 1), or in the intracellular tail (couple 2). In both the homozygote condition, or in transheterozygote with the null alleles, the Piggybac element led to a loss of more than 90% of transcripts. C) and D) Specificity of NMJ Dg staining observed with the anti-Dgex8 antibody on muscle 4 NMJs. Dgex8 (panels 1 and 2) or Discs-Large (panels 1 and 3) immunoreactivity in ywCS control (C) and dg1554/dg323 mutant larvae (D). The Dgex8 NMJ immunoreactivity almost completely disappears in the mutant condition whereas Discs-Large immunoreactivity is still present. Scale bar is 10 µm.
(2.12 MB TIF)

Figure S2 Dg-C-GFP can recruit Lam and Cora at extrasynaptic patches (A-C) Double staining against GFP (A, C) and Lam (A, B) in larvae overexpressing Dg-C-GFP in the muscles with the 24B Gal4 driver. In all panels of this figure, a single confocal optical section, which crosses the sarcolemma, is taken (D). The bottom right part of each panel corresponds the extracellular space and the up left part to the sarcoplasma. Lam colocalizes with Dg-C-GFP patches at the sarcolemma (see arrows in A). (E-G) Double staining against GFP (E, G) and Cora (E, F) in larvae overexpressing Dg-C-GFP in the muscles with the 24B Gal4 driver. Cora colocalizes with Dg-C-GFP patches at the sarcolemma (see arrows in E). (H-J) Double staining against Cora (H, I) and Lam (H, J) in wild-type larvae, without any Dg overexpression. There are no extrasynaptic patches of Cora and Lam in these larvae. Scale bar is 10 µm.
(1.40 MB TIF)

Figure S3 Cora is concentrated at the third instar larval NMJ and colocalizes with Spectrin. Double staining for Cora (red) and HRP (blue)(1) on (A) WT larvae, (B) cora14/corak08713 mutant larvae and (C) P{EPgy2}cora[EY07598]/+; 24B Gal4/+ larvae, which overexpress all Cora isoforms in muscles. Muscle 4 NMJs are shown. Single stainings for Cora and HRP are shown respectively in (2) and (3). Scale bar is 10 µm. (D) Immunoblot of proteins isolated from ywCS, cora14/corak08713 and P{EPgy2}cora[EY07598]/+; 24B Gal4/+ larvae. This blot was stained with the polyclonal Guinea-pig anti-Cora antibody. The wild-type larvae display 2 bands of about 210 and 240 kDa. The signal intensity is clearly reduced in cora hypomorph mutants, whereas it is enhanced when Cora is overexpressed with the 24B-Gal4 driver. A second staining of the same blot with an anti-Tubulin antibody indicated that the protein loading was similar in all lanes. Relative molecular mass size markers (kDa) are indicated at right. (E) Triple staining for HRP (blue), Cora (red) and alpha-Spectrin (green) on WT third instar larvae (E1). A muscle 4 NMJ is shown. Single stainings for HRP, Cora and alpha-Spectrin are displayed respectively in E2, E3 and E4. A plot of the intensity of each labelling along a line crossing a terminal bouton (see in E1) is represented in E5. Note that the extent of alpha-Spectrin and Cora stainings around the synaptic bouton -labelled with HRP- is similar. Scale bar is 10 µm.
(5.67 MB TIF)

Figure S4 Model of protein interactions with Dg at the postsynaptic side of the Drosophila NMJ.
(0.67 MB TIF)
