# Supplementary figures and images for: Muscle Dystroglycan Organizes the Postsynapse and Regulates Presynaptic Neurotransmitter Release at the Drosophila Neuromuscular Junction
Source: PLoS One. 2008 Apr 30;3(4):e2084. doi: 10.1371/journal.pone.0002084 (PMC2323113; doi:10.1371/journal.pone.0002084)

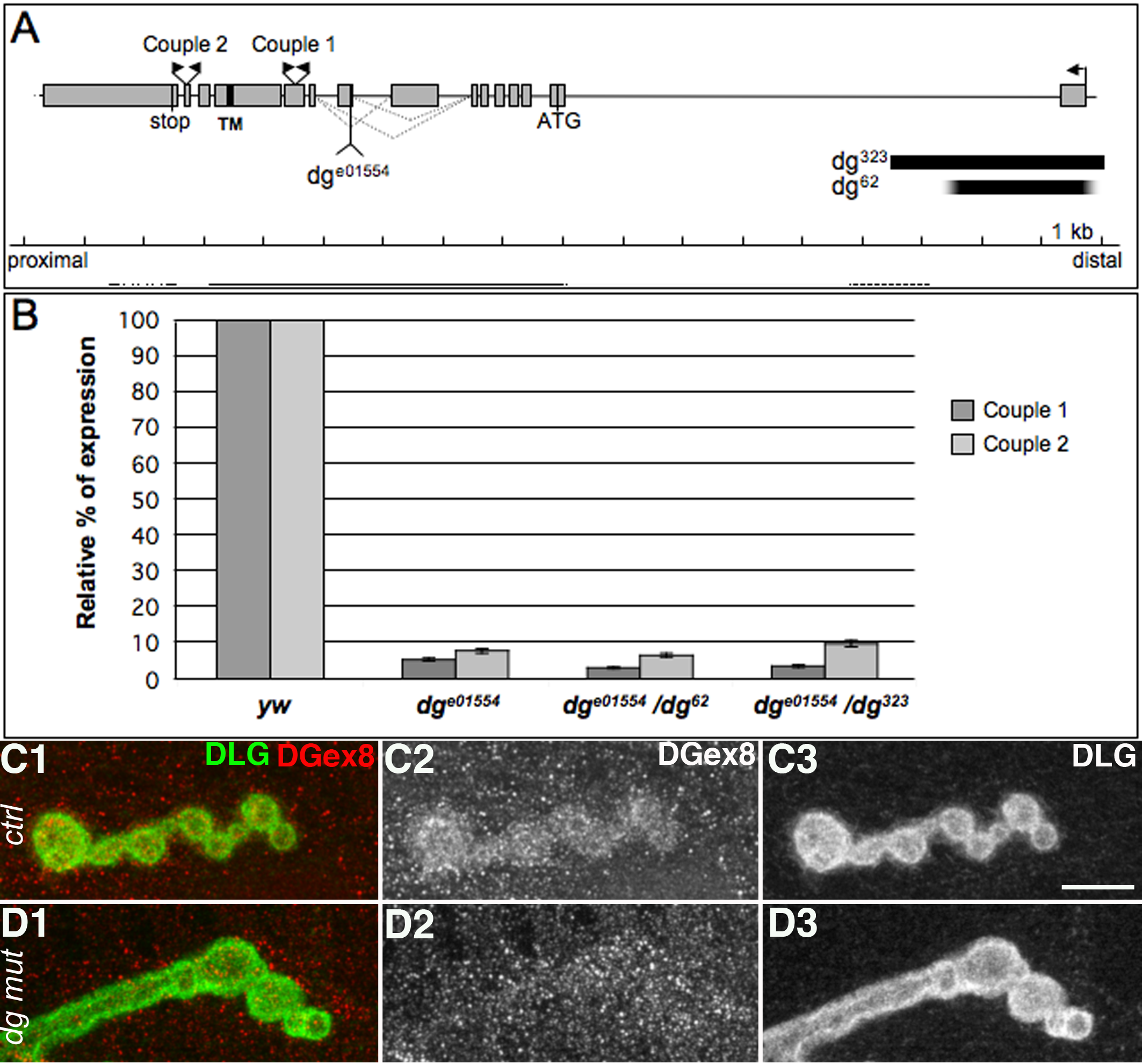

Supplement: Figure S1 — dg gene structure, dg mutants and qRT-PCR analysis A) Gene structure of dg gene compiled from flybase data and [27]. The two null alleles dg323 and dg62 are deletions comprising the first exon. The Piggybac element PBac{RB}e01554 is localized within the ninth exon, subjected to alternative splicing. B) To analyze how the Piggybac element altered dg transcription, we perfomed qRT-PCR experiments with couples of oligonucleotides designed against sequences common to all transcripts, either in the extracellular domain (couple 1), or in the intracellular tail (couple 2). In both the homozygote condition, or in transheterozygote with the null alleles, the Piggybac element led to a loss of more than 90% of transcripts. C) and D) Specificity of NMJ Dg staining observed with the anti-Dgex8 antibody on muscle 4 NMJs. Dgex8 (panels 1 and 2) or Discs-Large (panels 1 and 3) immunoreactivity in ywCS control (C) and dg1554/dg323 mutant larvae (D). The Dgex8 NMJ immunoreactivity almost completely disappears in the mutant condition whereas Discs-Large immunoreactivity is still present. Scale bar is 10 µm. (2.12 MB TIF) [file pone.0002084.s001.tif]

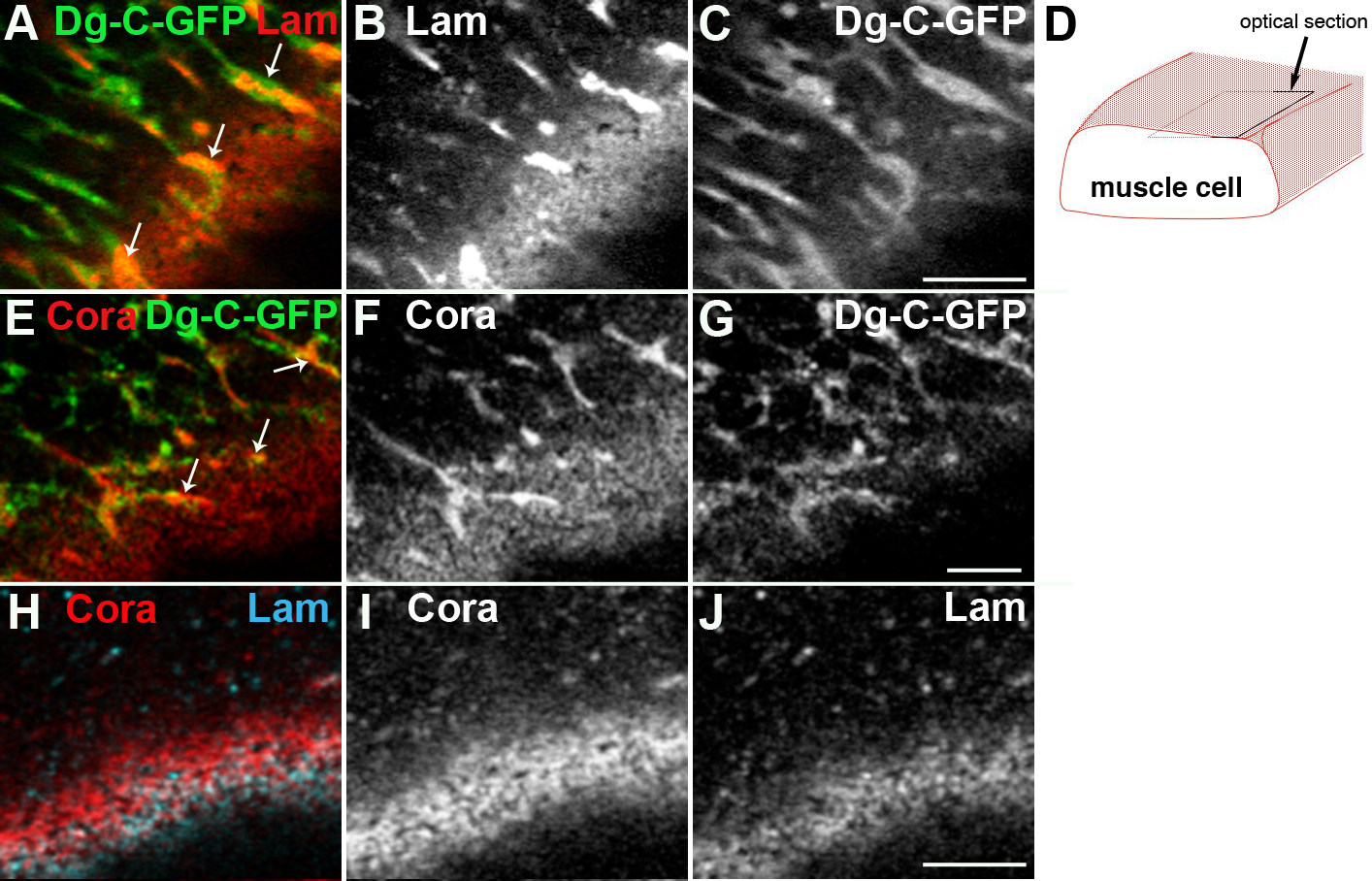

Supplement: Figure S2 — Dg-C-GFP can recruit Lam and Cora at extrasynaptic patches (A-C) Double staining against GFP (A, C) and Lam (A, B) in larvae overexpressing Dg-C-GFP in the muscles with the 24B Gal4 driver. In all panels of this figure, a single confocal optical section, which crosses the sarcolemma, is taken (D). The bottom right part of each panel corresponds the extracellular space and the up left part to the sarcoplasma. Lam colocalizes with Dg-C-GFP patches at the sarcolemma (see arrows in A). (E-G) Double staining against GFP (E, G) and Cora (E, F) in larvae overexpressing Dg-C-GFP in the muscles with the 24B Gal4 driver. Cora colocalizes with Dg-C-GFP patches at the sarcolemma (see arrows in E). (H-J) Double staining against Cora (H, I) and Lam (H, J) in wild-type larvae, without any Dg overexpression. There are no extrasynaptic patches of Cora and Lam in these larvae. Scale bar is 10 µm. (1.40 MB TIF) [file pone.0002084.s002.tif]

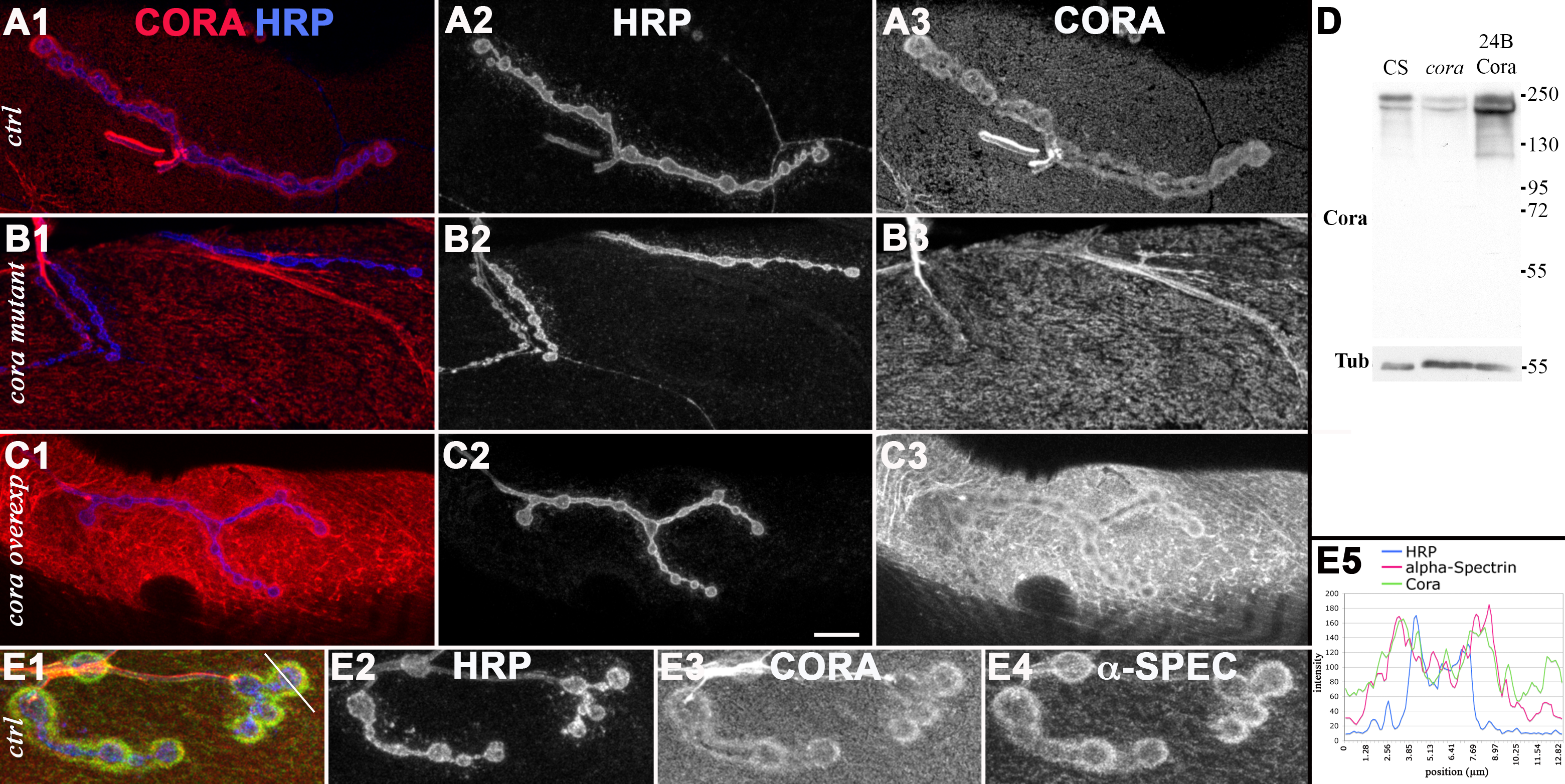

Supplement: Figure S3 — Cora is concentrated at the third instar larval NMJ and colocalizes with Spectrin. Double staining for Cora (red) and HRP (blue)(1) on (A) WT larvae, (B) cora14/corak08713 mutant larvae and (C) P{EPgy2}cora[EY07598]/+; 24B Gal4/+ larvae, which overexpress all Cora isoforms in muscles. Muscle 4 NMJs are shown. Single stainings for Cora and HRP are shown respectively in (2) and (3). Scale bar is 10 µm. (D) Immunoblot of proteins isolated from ywCS, cora14/corak08713 and P{EPgy2}cora[EY07598]/+; 24B Gal4/+ larvae. This blot was stained with the polyclonal Guinea-pig anti-Cora antibody. The wild-type larvae display 2 bands of about 210 and 240 kDa. The signal intensity is clearly reduced in cora hypomorph mutants, whereas it is enhanced when Cora is overexpressed with the 24B-Gal4 driver. A second staining of the same blot with an anti-Tubulin antibody indicated that the protein loading was similar in all lanes. Relative molecular mass size markers (kDa) are indicated at right. (E) Triple staining for HRP (blue), Cora (red) and alpha-Spectrin (green) on WT third instar larvae (E1). A muscle 4 NMJ is shown. Single stainings for HRP, Cora and alpha-Spectrin are displayed respectively in E2, E3 and E4. A plot of the intensity of each labelling along a line crossing a terminal bouton (see in E1) is represented in E5. Note that the extent of alpha-Spectrin and Cora stainings around the synaptic bouton -labelled with HRP- is similar. Scale bar is 10 µm. (5.67 MB TIF) [file pone.0002084.s003.tif]

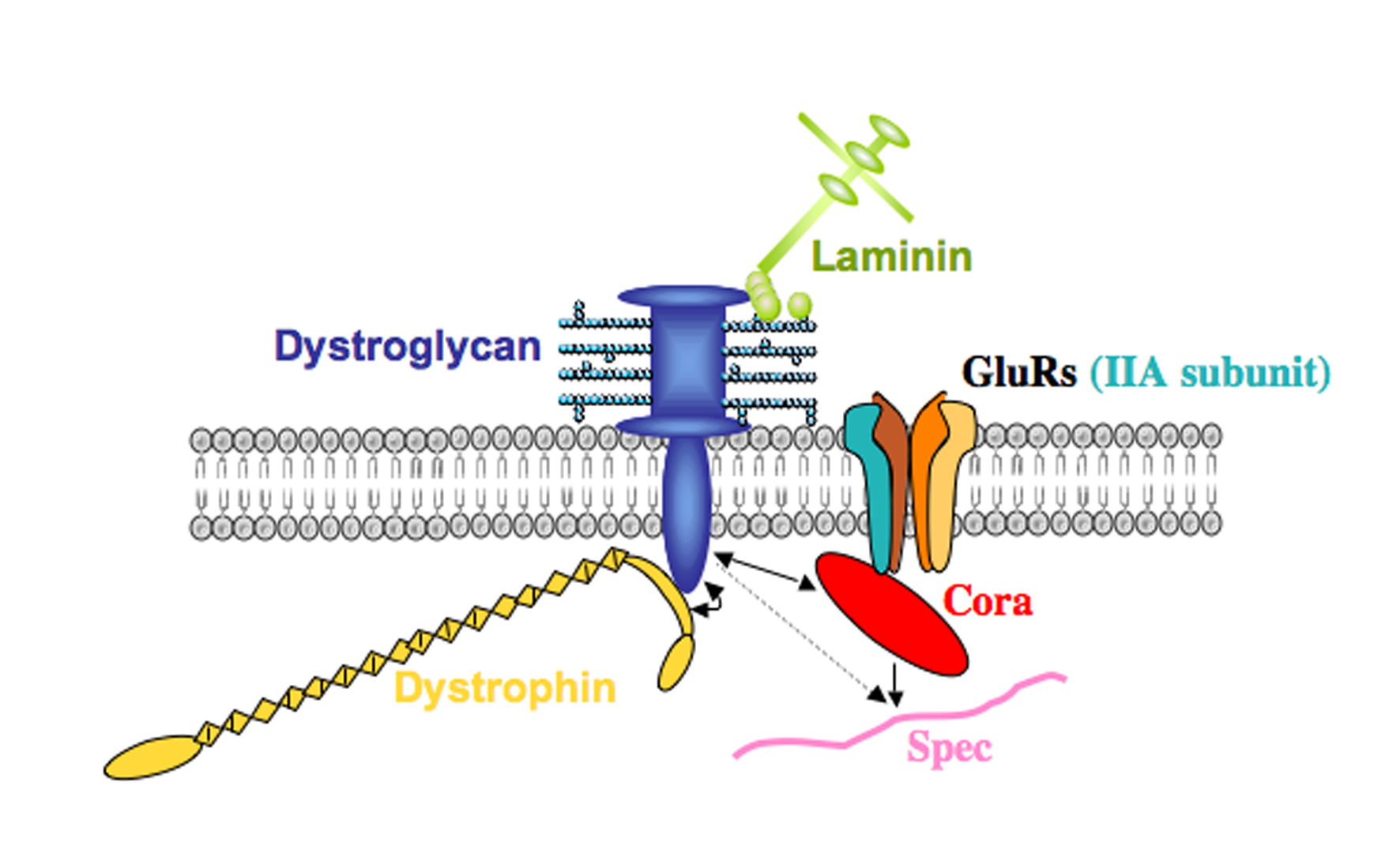

Supplement: Figure S4 — Model of protein interactions with Dg at the postsynaptic side of the Drosophila NMJ. (0.67 MB TIF) [file pone.0002084.s004.tif]
